# Myocarditis following mRNA vaccination against SARS-CoV-2, a case series

**DOI:** 10.1016/j.ahjo.2021.100042

**Published:** 2021-08-09

**Authors:** William W. King, Matthew R. Petersen, Ralph M. Matar, Jeffery B. Budweg, Lyda Cuervo Pardo, John W. Petersen

**Affiliations:** a Department of Internal Medicine, University of Florida, 1600 SW Archer Road, Room 4102, Gainesville, FL 32610-0277, United States of America; b Division of Cardiovascular Medicine, University of Florida, Gainesville, FL, United States of America; c Division of Rheumatology and Clinical Immunology, University of Florida, Gainesville, FL, United States of America

**Keywords:** COVID-19, Myopericarditis, Myocarditis, Pericarditis, mRNA vaccine

## Abstract

**Introduction:**

mRNA COVID-19 vaccines have emerged as a new form of vaccination that has proven to be highly safe and effective against COVID-19 vaccination. Rare adverse events including myocarditis have been reported in the literature.

**Methods:**

Data were gathered from the electronic medical record of four patients personally treated by the authors.

**Results:**

Four patients, ages 20 to 30, presented with myocarditis characterized by chest pain, elevations in troponin-I and C-reactive protein, and negative viral serologies two to four days following mRNA vaccine administration. One had a cardiac MRI showing delayed gadolinium enhancement in a subpericardial pattern. All experienced symptom resolution by the following day, and the two who have returned for follow-up had normal troponin-I and CRP values.

**Discussion:**

Along with previously reported instances, these cases raise suspicion for a possible link between mRNA vaccines and myocarditis.

## 1 Introduction

In 2020 SARS-CoV-2 spread across the globe, inducing hypoxic respiratory failure, acute respiratory distress syndrome, hypercoagulability, and severe systemic inflammation. Cardiovascular manifestations of COVID-19, the disease caused by SARS-CoV-2, include myocardial infarction, transient systolic and diastolic dysfunction, and myocarditis. Both preexisting cardiovascular disease and COVID-induced myocarditis are associated with higher mortality.

Myopericarditis refers to simultaneous myocarditis and pericarditis, inflammatory conditions of the myocardium and pericardium, respectively. Characteristic symptoms and objective findings raise suspicion for myocarditis. Symptoms include chest pain, dyspnea on exertion, palpitations, and unexplained cardiogenic shock. Objective findings include arrhythmias, conduction delays, troponin-I elevations, and functional or structural abnormalities on cardiac imaging. Often cardiac magnetic resonance imaging (CMR) demonstrates characteristic late gadolinium enhancement, supporting this diagnosis. However, definitive diagnosis requires endomyocardial biopsy, the sensitivity of which is low due to the focal and transient nature of infiltrates. Pericarditis is diagnosed by the presence of two or more of the following: pleuritic chest pain, pericardial friction rub, new pericardial effusion, and ECG changes including down-sloping PR depression and diffuse ST elevations.

In early 2020, researchers developed vaccines against SARS-CoV-2 utilizing a novel vaccination strategy of inoculating liposome-encapsulated recombinant mRNA encoding the SARS-CoV-2 spike protein. Phase 3 multicenter randomized controlled trials showed 94–95% efficacy in prevention of severe COVID-19 [1], [2]. In the Moderna trial, 1.5% of vaccine recipients and 1.3% of placebo recipients reported grade 3 adverse reactions, side effects altering daily activity. Similar numbers were reported in the Pfizer trial, with 1.2% of vaccine recipients and 0.7% of placebo recipients reporting severe adverse events. In both trials the most common systemic reactions were fatigue, headache, muscle pain, and chills. These effects occurred most frequently after the second dose and in participants in the youngest age group. They resolved on average 2–3 days post-vaccine. Neither study reported major cardiovascular adverse events, including myocarditis [1], [2]. Due to the efficacy and safety demonstrated in these clinical trials, the Food and Drug Administration granted emergency use authorization to both mRNA vaccines in December 2020.

## 2 Case 1

**Fig. 1:**
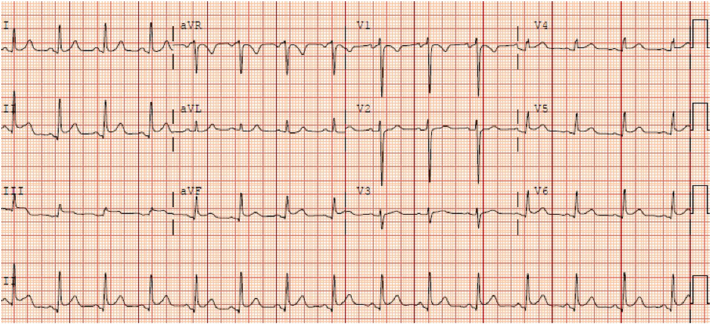
ECG with down-sloping PR depressions and diffuse ST elevations not following a single coronary distribution.

**Fig. 2:**
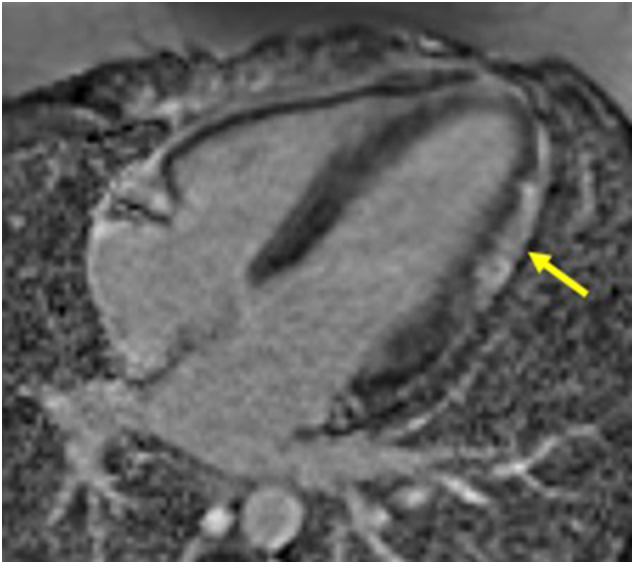
Four-chamber delayed enhancement phase sensitive inversion recovery sequence showing delayed gadolinium enhancement suggestive of fibrosis involving the mid to apical anterolateral wall segments (yellow arrow) in a subepicardial pattern of distribution consistent with myocarditis.

**Fig. 3:**
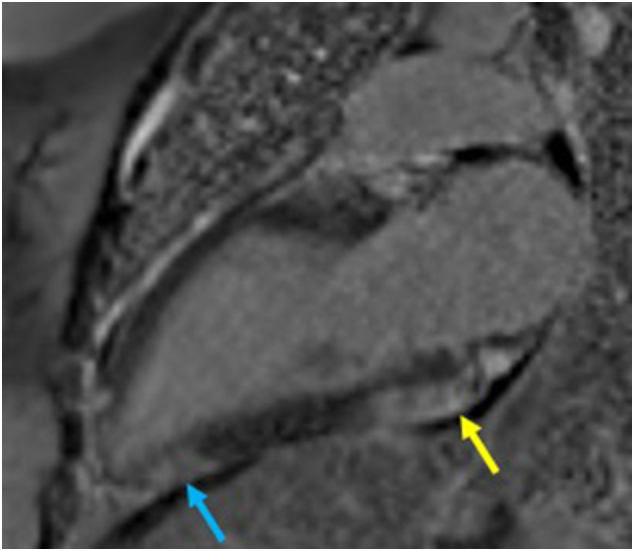
Two-chamber delayed enhancement phase sensitive inversion recovery sequence showing delayed gadolinium enhancement suggestive of fibrosis involving the basal inferior (blue arrow) and apical inferior (red arrow) wall segments in a subepicardial pattern of distribution consistent with myocarditis.

**Fig. 4:**
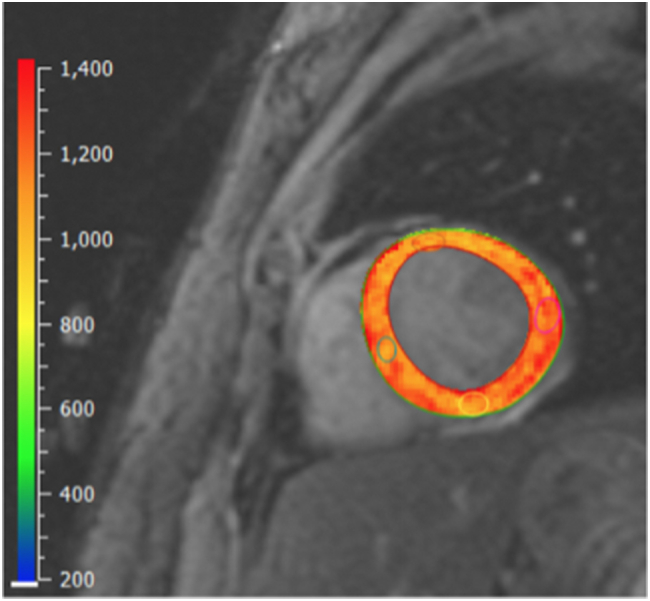
T1 mapping short-axis view showing diffusely elevated T1 relaxation times, most prominently in the lateral and septal wall segments.

A 23-year-old woman presented with chest pain 5 days after receiving her second dose of the Moderna vaccine. She had an ECG (Fig. 1) with down-sloping PR depressions and diffuse ST elevations, as well as a troponin of 14,045 pg/mL and an elevated CRP (Table 1). Her troponin peaked the following day. Coxsackie, HCV, CMV, and EBV serologies were all negative. Transthoracic echocardiography (TTE) demonstrated a left ventricular ejection fraction (LVEF) of 55 to 60%, with basal inferior and basal inferolateral hypokinesis. CMR (Fig. 2, Fig. 3, Fig. 4) revealed late gadolinium enhancement involving the basal inferior, basal to mid inferolateral, mid anterolateral, apical lateral, apical septal, and apical inferior wall segments in a subepicardial distribution pattern, consistent with myocarditis. Her symptoms resolved quickly, and her CRP declined to 11 mg/L by the third day of her hospitalization. She was discharged on hospital day 3. She presented to clinic for follow-up two weeks after discharge, where her CRP had declined to 0.8 mg/L and she had no residual symptoms.

**Table 1:** Summary of clinical findings. All patients presented 2 to 5 days following their 2nd vaccine dose with troponin and CRP elevation, and the viral serologies that were tested were negative. ECG and TTE abnormalities may be compared as well.

Case number	Age and sex	Vaccine maker	Days from 2nd vaccine dose to presentation	Peak troponin-I (pg/mL)	CRP (mg/L)	Viral serologies^a^	ECG abnormalities	TTE findings
1	23F	Moderna	5	16,263	41	Negative	Down-sloping PR depressions, diffuse ST elevations	LVEF 55–60%, basal inferior and basal inferolateral hypokinesis
2	20M	Moderna	2	>27,000	88	Negative	Down-sloping PR depressions, diffuse ST elevations	LVEF 45%, apical septal hypokinesis
3	29M	Moderna	4	6802	14	–	Down-sloping PR depressions, diffuse ST elevations	LVEF 55%, no regional wall motion abnormalities
4	30M	Pfizer	4	2518	129	Negative	T-wave inversions in lateral leads	LVEF 65–70%, no regional wall motion abnormalities

CRP: C-reactive protein.

ECG: electrocardiogram.

TTE: transthoracic echocardiogram.

LVEF: left ventricular ejection fraction.

a Coxsackie virus, EBV, CMV.

## 3 Case 2

A 20-year-old man presented with a 2-day history of progressive chest pain, 2 days after receiving his second dose of the Moderna vaccine. His symptoms started with a viral prodrome approximately ten days prior to the onset of his chest pain. His ECG had down-sloping PR depressions and diffuse ST elevations; his troponin-I was 22,638 and CRP was markedly elevated (Table 1). Troponin-I peaked the following day. Viral serologies for HIV, hepatitis B and C viruses, coxsackie virus type b, and EBV were all undetectable. TTE revealed a LVEF of 45% with moderate hypokinesis of the apex and apical septum. Outpatient CMR remains pending. His chest pain resolved the following day. He was discharged on hospital day 3. He presented to clinic eleven days after discharge, where he his troponin had normalized to 0.03 ng/dL, and his CRP to 2.5 mg/L.

## 4 Case 3

A 29-year-old man presented with chest pain 4 days after receiving his second dose of the Moderna vaccine. His ECG had diffuse ST elevations with no PR depressions; initial troponin-I was 3785 pg/mL and CRP was notably elevated (Table 1). Troponin-I peaked the next day. TTE revealed and EF of 55% with no regional wall motion abnormalities. He did not undergo CMR or viral serology testing. An autoimmune workup showed an anti-nuclear antibody titer of 1:80 in a speckled pattern and negative double stranded DNA, rheumatoid factor, ribonucleic protein IgG, scleroderma-70, anti–Sjögren's-syndrome-related antigen A, and anti-Smith autoantibodies were negative. His chest pain resolved on the first day of his hospitalization, and he was discharged the following day.

## 5 Case 4

A 30-year-old man presented on with chest pain 4 days after receiving his second dose of the Pfizer vaccine. ECG was notable only for T-wave inversions in the lateral leads that resolved on follow-up ECG. Troponin-I was 2447 pg/mL and CRP was notably elevated (Table 1). Troponin-I peaked the next day. EBV, CMV, and coxsackie serologies were all negative. CMR was not performed. TTE was unremarkable with normal LVEF and no regional wall motion abnormalities. His symptoms resolved on first day of hospitalization, and he was discharged on hospital day 3.

## 6 Discussion

This is among the first series to report multiple cases of myocarditis in adults following vaccination against SARS-CoV-2. All four patients were young, between 20 and 30. All presented with chest pain two to five days after their second vaccine dose. All had significantly elevated troponin-I levels. Though one had a viral prodrome, all had negative serologies. None reported prior COVID-19 infection. None had stigmata of autoimmune disease, and the one who underwent a rheumatologic workup while hospitalized had unremarkable autoimmune serologies. Reassuringly, the two patients who have returned for follow up in the weeks following discharge had normalized CRP values and denied symptom recurrence.

Myocarditis is most often caused by direct viral injury or by autoimmune mechanisms but has been sporadically linked to vaccination. Over 50 cases had been reported to the Department of Defense Smallpox Vaccination Program [3]. Myopericarditis has also been reported soon after vaccines against anthrax, *haemophilus influenzae* type b, hepatitis B virus, inactivated influenza, and live attenuated zoster vaccines [4].

Neither clinical trial reported adverse cardiac events including myocarditis [1], [2]. As vaccination rates increase among younger patients, however, several cases of post-vaccine myocarditis are being reported in adolescents and young adults [5], [6], [7]. In addition to these anecdotes, a multinational cohort study analyzed electronic health record databases and found the incidence of myocarditis and pericarditis among vaccine recipients aged 18 to 35 to be approximately 0.016% for women and 0.037% for men [8]. The CDC has since warned clinicians to be wary of post-vaccine myocarditis in teens and young adults [9]. It remains unclear why younger patients are more prone to develop this adverse effect. A possible explanation could be related to the stronger immune response in younger patients, which can also explain the higher prevalence of side effects to the vaccines in this patient population [10].

While certainly a pattern worth exploring, this case series has numerous limitations, including a small sample size, variation in workup and treatment strategies, and retrospective analysis insufficient to establish causality. Nevertheless, the odds of incidental seronegative viral myocarditis occurring in four patients presenting to a single medical center within days of vaccine administration would be long. The authors would encourage further investigation and reporting of potential cases of post-vaccine myocarditis. The authors seek not to frustrate vaccination efforts, but rather to prepare patients and providers for a rare but potential adverse effect. Furthermore, the authors hope the dramatic improvement in all four patients will reassure those who do suffer from myocarditis following vaccination.

## Declaration of competing interest

The authors declare that they have no known competing financial interests or personal relationships that could have appeared to influence the work reported in this paper.
